# Hypervalent Iodine-Catalyzed Fluorination of Diene-Containing Compounds: A Computational Study

**DOI:** 10.3390/molecules29133104

**Published:** 2024-06-29

**Authors:** Tianci Liu, Hai-Bei Li

**Affiliations:** 1 SDU-ANU Joint Science College, Shandong University, Weihai 264209, China; 202100700188@mail.sdu.edu.cn; 2 Marine College, Shandong University, Weihai 264209, China

**Keywords:** fluorination, hypervalent iodine, reaction mechanism

## Abstract

Studies have shown that the incorporation of fluorine into materials can improve their properties, but C–F bonds are not readily formed in nature. Although some researchers have studied the reaction of fluorinating alkenes catalyzed by hypervalent iodine, far too little attention has been paid to its reaction mechanism. This study aimed to explore the mechanism of the hypervalent iodine-catalyzed 1,4-difluorination of dienes. We found that the catalyst is favorable for the activation of C1=C2 double bonds through halogen bonds, and then two HFs interact with one F atom in the catalyst via hydrogen bonds, resulting in the cleavage of I–F bonds and the formation of [F–H∙∙∙F]^−^. Subsequently, the catalyst interacts with C1, and the roaming [F–H···F]^−^ attacks C4 from the opposite side of the catalyst. After the fluorination step is completed, the nucleophile F^−^ substitutes the catalyst via the S_N_2 mechanism. Our calculations demonstrated that the interaction between HF and F^−^ is favorable for the stabilization of the transition state within the fluorination process for which the presence of two HFs in the reaction is the best. We also observed that [F–H∙∙∙F]^−^ attacking C4 from the opposite side of the catalyst is more advantageous than attacking from the same side. This study therefore offers a novel perspective on the mechanism of the hypervalent iodine-catalyzed fluoridation of dienes.

## 1. Introduction

Previous studies have demonstrated that the C–F bond is one of the strongest known bonds [1,2]. Moreover, incorporating fluorine atoms into materials can affect their physicochemical properties, such as their lipophilicity, metabolic stability, and bioavailability [3]. Therefore, increasing emphasis has been directed toward developing new synthetic methods for the incorporation of fluorine-containing substituents into organic molecules. However, the formation of C–F bonds in nature is rare, and fluorine-containing compounds are mostly artificially synthesized [3,4,5].

Organofluorine compounds have a wide range of applications in medicine, pesticides, materials science, and other fields [6]. Fluorine atoms can change the properties of a compound and enhance its drug-binding efficiency and selectivity [7]. At present, approximately 20% of commercial drugs are fluorinated [8]. Many marketed pharmaceuticals, such as anticancer medications, antibiotics, and antiviral drugs, contain one or more fluorine atoms [9]. Fluorine also has a dramatic effect on the biological activity of agrochemicals [10]. Many fluorine compound structures are used to control pests and diseases, thus improving crop yield [11]. In addition, due to the strength of C–F bonds, fluorine-containing compounds have higher thermal and oxidative stability, lower polarity, and lower surface tension than hydrocarbons [12,13,14]. Therefore, fluorine-containing compounds are extensively used in materials engineering [13]. For example, energetic materials that contain trifluoromethyl functional groups have the advantages of high density and high thermal stability [15]. Because organic fluorides are widely used, their preparation has become a popular and significant research topic.

Due to the challenges of energy scarcity and environmental pollution, the search for clean energy sources and environmentally friendly catalysts is of paramount importance [16]. Hypervalent iodine catalysts can meet the requirements of environmental protection. Compared with traditional transition metal catalysts, hypervalent iodine catalysts generally have lower toxicity and higher bioavailability [17,18]. Existing studies have also established the remarkable catalytic effect of hypervalent iodine within various reactions [19]. Under mild conditions, hypervalent iodine catalysts can catalyze various organic reactions, such as oxidation [20], water-mediated reactions [21], and carbon–carbon bond formation [22]. In oxidation reactions, it can provide an appropriate chiral environment for enantioselective transformations. In water-mediated reactions, water molecules can coordinate with the iodine in the catalyst, which helps to enhance their thermal stability. Additionally, significant progress has been made in C-C bond formation through hypervalent iodine-mediated electrophilic substitution reactions, arylations, and photooxidation–reduction reactions. Reactions using hypervalent iodine as a catalyst are usually characterized by high efficiency, high selectivity, and high yield. In recent years, the functionalization of olefins has attracted widespread attention, and transition metal catalysts play an important role in olefin functionalization reactions. For example, rhodium [4] and copper [5] are widely used to catalyze the functionalization of alkenes. However, hypervalent iodine catalysts have obvious advantages over transition metal catalysts [23]. Studies that have used hypervalent iodine reagents in organic synthesis reveal that hypervalent iodine reagents usually involve simple operation, mild reaction conditions, and good selectivity [24,25]. Therefore, hypervalent iodine catalysts hold promise for the construction of organic fluorides.

**Figure 1:**
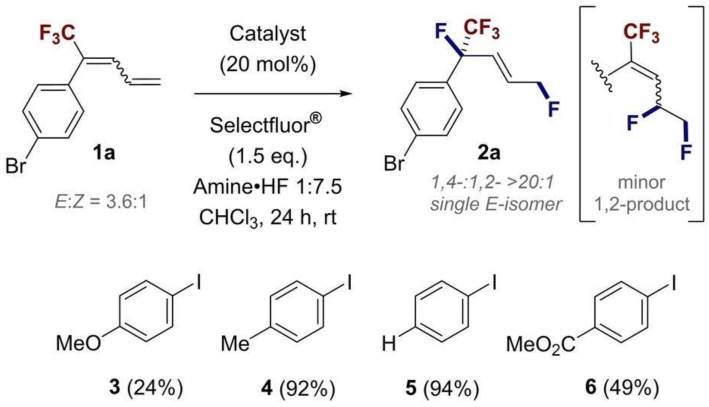
Fluorination reaction of 1,4-dienes with hypervalent iodine reagents [29].

Most existing research on the mechanisms of organic catalysis has focused on transition metal catalysts instead of hypervalent iodine catalysts. Although there have been some studies on the fluorination of alkenes catalyzed by hypervalent iodine [26,27,28], research on hypervalent iodine agents that catalyze dienes is still scarce. Through experiment, Yu et al. proposed a method for fluorinating 1,4-dienes using hypervalent iodine reagents as catalysts [29] (Figure 1). On this basis, in this study, we investigated the mechanism of catalyzing dienes using hypervalent iodine catalysts. The catalyst activates a specific double bond in dienes through a halogen bond, thus forming a complex. Then, two HFs interact with one F atom in the catalyst, leading to the cleavage of the I–F bond and the formation of one roaming [F–H···F]^−^ in the system. Subsequently, the roaming [F–H···F]^−^ attacks C4 from the opposite side of the catalyst, rather than from the same side, leading to the formation of a C–F bond. Finally, the nucleophile F^−^ attacks C1 via the S_N_2 mechanism, yielding the difluorinated product. We found that the presence of two HFs is the optimal solution for reducing activation energy barrier in the fluorination step. This study greatly improves our understanding of the selectivity and mechanism of catalysis involving hypervalent iodine agents. It also provides new ideas and methods for the application of hypervalent iodine catalysts in the field of organic synthesis.

## 2. Results

### 2.1. Electrostatic Potential Analysis

**Figure 2:**
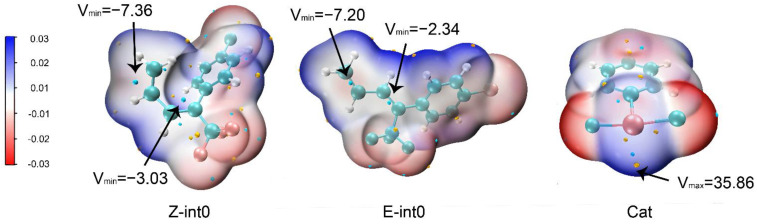
Electrostatic potential (ESP) maps on the 0.001 a.u. the contour of the electronic density of the reactants Z-int0 and E-int0, as well as the hypervalent iodine catalyst. The color ranges in the map are in kcal/mol. Blue corresponds to the maximum ESP value, while red corresponds to the minimum ESP value; intermediate colors correspond to intermediate values of ESP. The local extremes are indicated by arrows.

Electrostatic potential is a good predictor of chemical reactivity and molecular interactions [30,31]. Regions with positive electrostatic potential are susceptible to attack by regions with negative electrostatic potential. Figure 2 shows an electrostatic potential diagram of the catalyst and Z/E-dienes. The local maximum of electrostatic potential is observed around the I atom, which is 35.86 kcal/mol. On the other hand, for Z/E-dienes, there are local minima of electrostatic potential at the position of the double bond. It is evident from Figure 2 that sites with a small steric hindrance have more negative electrostatic potential than sites with a large steric hindrance. The results of the electrostatic potential diagram show that the hypervalent iodine catalyst can interact with double bonds of Z/E-dienes through electrostatic interaction. Therefore, considering the electronic structures of this catalyst and Z/E-dienes, it is theoretically feasible to choose hypervalent iodine catalysts for the difluorination of dienes.

### 2.2. The Mechanism of Diene Difluoride

Due to their higher HOMO and lower LUMO levels, conjugated dienes are more reactive than isolated double bonds. In addition, Z-olefins and E-olefins have different thermodynamical stabilities, so their reaction processes are different when exposed to the same catalyst. Thus, the E/Z configuration should be considered when polyenes are used as reactants.

#### 2.2.1. Difluorination of E-Configured Dienes Catalyzed by Hypervalent Iodine Reagents

**Figure 3:**
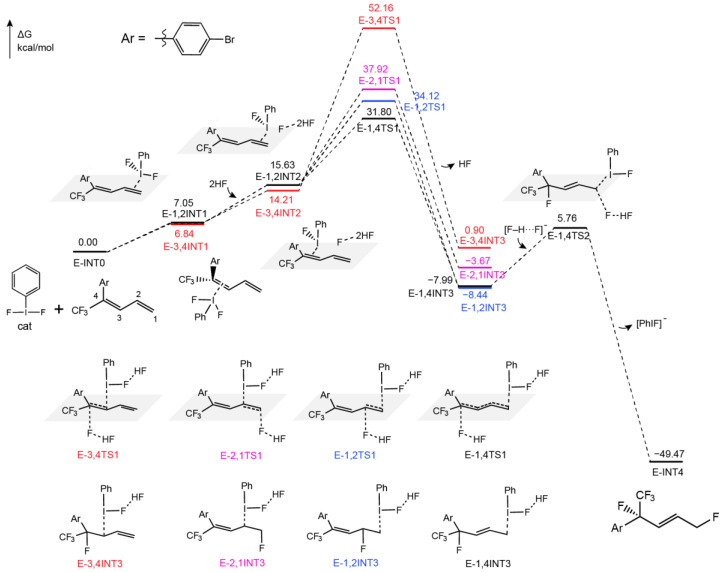
The mechanism of hypervalent iodine-catalyzed fluorination of dienes when the diene is in the E configuration. Free energies are given in kcal/mol at the M06-2X/6-311++G(d, p) and SDD(I)-SMD(CHCl_3_)//M06-2X/6-31G(d, p) and LANL2DZ(I) level of theory. The reaction was carried out at 298.15 K.

As discussed above, given the electrostatic potentials of the hypervalent iodine catalyst and E-dienes, it is feasible for the catalyst to activate the double bonds in E-diene via halogen bonds, thus forming intermediates E-1,2INT1 and E-3,4INT1. The Gibbs free energy of the complex increases by 7.05 kcal/mol for E-1,2INT1 and 6.84 kcal/mol for E-3,4INT1 (Figure 3). After the intermediates are formed, the F atoms in E-1,2INT1 and E-3,4INT1 interact with two HFs through hydrogen bonds, resulting in the cleavage of one of the I–F bonds. Subsequently, in E-1,2INT2, the catalyst activates the C1=C2 double bond; thus, it will interact with C1 or C2 in the fluorination step. When the catalyst interacts with C1, the roaming [F···H–F]^−^ can attack C2 or C4 from the opposite side of the catalyst, and the calculated energy barrier values are 18.49 kcal/mol and 16.17 kcal/mol, respectively. In a subsequent section, we will discuss the activation barrier height when the attack occurs from the same side of the catalyst. When the catalyst interacts with C2, [F···H–F]^−^ can only attack C1 from the opposite side of the catalyst; in this case, the activation energy barrier is 22.18 kcal/mol. In E-3,4INT2, the catalyst activates the double bond between C3 and C4. Due to the large steric hindrance, the catalyst can only interact with C3, and therefore [F···H–F]^−^ attacks C4. The energy barrier from E-3,4INT2 to E-3,4TS1 is 37.95 kcal/mol, which is higher than the other three pathways mentioned above. Thus, the addition of 1,4-difluoride via the transition state E-1,4TS1 is the most favorable pathway among these pathways.

After the fluorination product is formed, the substitution of the catalyst with a nucleophile such as F^−^ via the S_N_2 mechanism is straightforward. The Gibbs free energy barrier for this step is 13.75 kcal/mol (from E-1,4INT3 to E-1,4TS2, as shown in Figure 3). Therefore, 1,4-difluoride is the main product in the difluorination of E-diene catalyzed by hypervalent iodine reagents. 

#### 2.2.2. Difluorination of Z-Configured Dienes Catalyzed by Hypervalent Iodine Reagents

**Figure 4:**
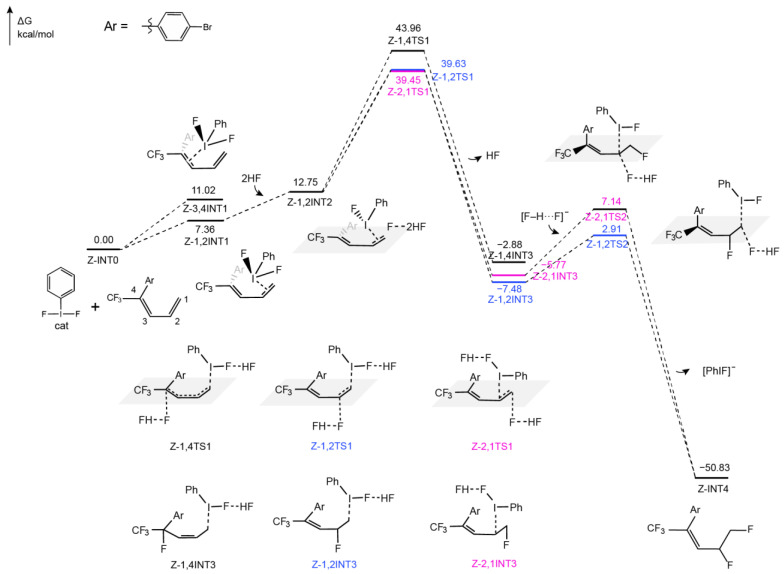
The mechanism of hypervalent iodine-catalyzed fluorination of dienes when the diene is in the Z configuration. Free energies are given in kcal/mol at the M06-2X/6-311++G (d, p) and SDD (I)-SMD(CHCl_3_)//M06-2X/6-31G (d, p) and LANL2DZ (I) level of theory. The reaction was carried out at 298.15 K.

Similarly, the first step also involves the formation of the complex (Z-1,2INT1 and Z-3,4INT1 in Figure 4) between the catalyst and Z-diene via halogen bonds. When the hypervalent iodine catalyst activates the C1=C2 double bond, the Gibbs free energy increases by 7.36 kcal/mol, whereas it is 11.02 kcal/mol as the catalyst activates the C3=C4 double bond, which is larger. As a result, we do not take 3,4-activation into account in the following steps. As with E-1,2INT1, after the activation of the double bond, Z-1,2INT1 is generated. Then, one F atom in the catalyst forms a hydrogen bond with two HFs, resulting in the cleavage of the I–F bond and the formation of roaming [F–H···F]^−^. Subsequently, the I atom in the catalyst interacts with C1 or C2. As the catalyst interacts with C1, the roaming [F–H···F]^−^ attacks C2 or C4 from the opposite side of the catalyst, in which case the activation energy barriers are 26.88 kcal/mol and 31.21 kcal/mol, respectively. When the catalyst interacts with C2, [F–H···F]^−^ attacks C1. The calculated Gibbs free energy is 26.70 kcal/mol (Figure 4). Different with that in E-diene, 1,2-fluoride is more favorable than that of 1,4-fluoride. As a result, after the fluorination product is developed, the nucleophile F^−^ attacks C1 or C2 via the S_N_2 mechanism, forming 1,2-difluoride products with activation energy barriers of 10.39 kcal/mol and 14.63 kcal/mol, respectively (Figure 4).

Taken together, the fluorination process is the rate-determining step within the whole reaction for the difluorination of both E- and Z-dienes. A comparison of the fluorination of E-diene and Z-diene reveals that the formation of E-configured 1,4-fluoride is more favorable than that of E/Z-configured 1,2-fluoride due to a lower activation energy barrier. The energy differences of ΔH and ΔS were also calculated in this fluorination process (see the Supplementary File, Table S1). We found that the entropy effect is more significant in Z-diene than in E-diene (Table S1), which illustrates that the entropy effect plays a crucial role in the formation of the main product of E-configured 1,4-fluoride experimentally. This proposed mechanism well explains why 1,4-fluoride is the main product in the experimental product [29]. According to the calculation, the activation energy barrier of the optimal path is 16.17 kcal/mol, which proves that the reaction can occur at room temperature. This is also consistent with the experimental reaction conditions [29]. 

**Figure 5:**
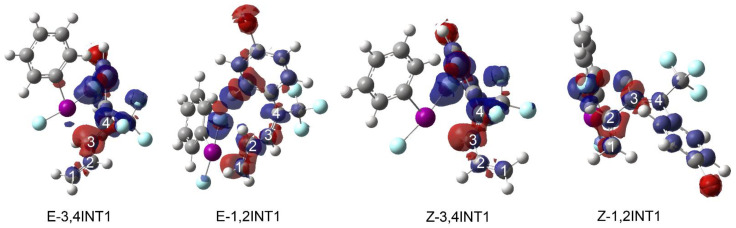
The density difference diagrams for the intermediates E-3,4INT1, E-1,2INT1, Z-3,4INT1, and Z-1,2INT1. The blue region represents a negative region of reduced electron density, and the red region represents a positive region of increased electron density.

In this reaction mechanism, the hypervalent iodine catalyst plays an important role. We explored the differences in density after the catalyst interacted with E/Z-diene to understand the role of the catalyst. The presence of the catalyst led to the electron transfer on the conjugated diene backbone. It can be seen from Figure 5 that the electron density of the C atom in double bonds closest to the iodine atom increases, while the electron density of other carbons on the double bonds decreases to some extent. Since the next step is F^−^ attacking C, the decrease in electron density on the C atom is beneficial. 

### 2.3. The Role of HF

**Figure 6:**
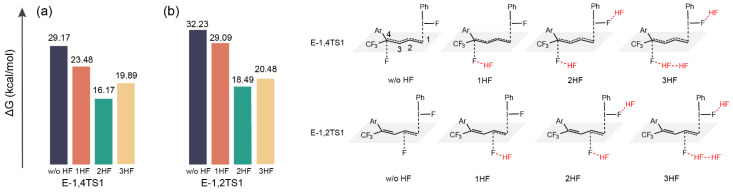
The difference of the activation energy barrier (ΔG) for TS in the fluorination process without HF, TS with one HF, TS with two HFs, and TS with three HFs: (**a**) F^−^ attacks C4 from the opposite side of the catalyst in the E configuration; (**b**) F^−^ attacks C2 from the opposite side in the E configuration.

There is a large number of HF molecules in the reaction system, which facilitates their interaction with the F atom in the catalyst by forming hydrogen bonds. Our calculations showed that the interaction between HFs and the F atom in the catalyst is favorable for the formation of the roaming [F–H···F]^−^ (Figure 3 and Figure 4). It also stabilizes the transition state during the fluorination of E-diene and F− attack. Figure 6 shows the difference in activation energy barrier heights (ΔG) of the fluorination step when different amounts of HF are present, including without HF, with one HF, two HFs, and three HFs. It can be seen from Figure 6 that as HFs (including one, two, and three HFs) form the roaming [F–H···F]^−^ with F^−^, the activation energy barrier in the fluorination process becomes lower than that without the assistance of HF. Due to the entropy effect, the participation of more HF molecules in the reaction is not conducive to the reaction. Thus, the presence of two HFs is optimal for reducing the activation energy barrier of the fluorination process of E-diene.

### 2.4. The Direction of Preference in F^−^ Attacking C 

**Figure 7:**
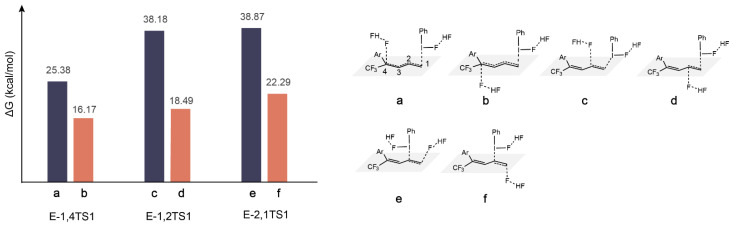
The activation energy barrier (ΔG) of E-TS1 in the fluorination process when F^−^ attacks from the same side of the catalyst as well as from the opposite side.

There are two directions from which [F–H···F]^−^ can attack the C atom on the double bond of diene: from the same side or the opposite side of the catalyst. We calculated the activation energy barrier heights for both cases. It is evident from Figure 7 that the energy barrier of fluorination is lower when F^−^ attacks from the opposite side of the catalyst than from the same side. We attribute this to the higher electronegativity of F compared to I, which causes I to be positively charged. As a result, the I atom can interact with the roaming F^−^, which hinders F^−^ from attacking C. This is confirmed by the distances between F^−^ and I in the geometries of a (2.65 Å) and b (6.24 Å) in Figure 7. 

## 3. Materials and Methods

All density functional calculations were performed using the Gaussian 16 program [32]. Geometry optimization and frequency calculations were performed with the M06-2X functional [33] using the mixed basis sets, namely LANL2DZ [34] for iodine atoms and 6-31G (d, p) basis sets for other atoms. The M06-2X functional is a high-nonlocality functional with double the amount of nonlocal exchange (2X), which has been frequently used related to hypervalent iodine catalysts [35,36] and non-covalent interactions [33]. Frequency calculations showed that the intermediate had no imaginary frequency, and the transition states had one imaginary frequency. The SMD solvation model [37] and M06-2X function were used to calculate the higher-level single-point energy. The SDD basis set was used for analyzing the iodine atom [38], and other atoms were analyzed with the 6-311++G (d, p) basis set.

The electrostatic potentials of the reactants and catalysts were calculated on the 0.001 au isosurface of the electron density, and a diagram was drawn showing electron density differences. This was accomplished by analyzing the changes in electrons using the density difference method. The above analysis was performed using Multiwfn 3.8 [39] and GaussView 06 software [40].

## 4. Conclusions

In this study, the role of hypervalent iodine catalysts in diene fluorination was investigated using density functional theory calculations. In the catalyst, the interaction between the iodine atom and two fluorine atoms makes iodine electropositive, allowing it to form a halogen bond with the double bonds of the diene. After the diene forms a complex with a hypervalent iodine reagent, one F atom in the catalyst forms hydrogen bonds with two HFs in the solvent, leaving the I atom, which leads to the formation of the roaming [F–H∙∙∙F]^−^. Then, the roaming [F–H∙∙∙F]^−^ attacks C4 from the opposite side of the catalyst, following the formation of 1,4-difluoride as a result of nucleophile F^−^ attacking C1 via the S_N_2 mechanism. We found that during fluorination step, attacking C from the opposite side of the catalyst leads to a lower energy barrier than attacking it from the same side. Furthermore, the reaction energy barrier markedly decreases due to the presence of two HFs in the reaction. The mechanism proposed in this study can provide a better understanding of the underlying mechanism of diene fluorination using hypervalent iodine as the catalyst.

## Data Availability

The data relevant to this article are contained within the article and the Supplementary Material. Further inquiries can be directed to the corresponding author.

## Supplementary Materials

The following supporting information can be downloaded at: https://www.mdpi.com/article/10.3390/molecules29133104/s1, Table S1: Thermodynamic parameters of the transition state in the fluorination step at 298K (all units are in kcal/mol).
