# Supplementary material for: Hypervalent Iodine-Catalyzed Fluorination of Diene-Containing Compounds: A Computational Study
Source: Molecules. 2024 Jun 29;29(13):3104. doi: 10.3390/molecules29133104 (PMC11243597; doi:10.3390/molecules29133104)
Supplement: Supplementary file 1 [file molecules-29-03104-s001.zip › molecules-3052415-supplementary.pdf]

Supporting Information for

"Hypervalent iodine-catalyzed fluorination of diene-containing compounds: a computational study"

Tianci Liu [1] and Hai-Bei Li [1,2,*]

Contents:

1. Table S1. Thermodynamic parameters of the transition state in the fluorination step at 298K (all units are in kcal/mol)

2. The coordinates of the geometries in the context.

1.Table S1. Thermodynamic parameters of the transition state in the fluorination step at 298K (all units are in kcal/mol)

Species	ΔH	ΔS (×10^{-3})	ΔG
E-14TS1	15.55	-2.07	16.17
E-12TS1	18.31	-0.65	18.49
E-21TS1	21.25	-3.48	22.29
E-34TS1	33.82	-13.82	36.53
Z-21TS1	21.34	-17.97	26.70
Z-12TS1	20.52	-21.31	26.88
Z-14TS1	21.94	-31.09	31.21

2.The coordinates of the geometries in the context.

Cartesian coordinates of HF

F	0.00000000	0.00000000	0.09211200
H	0.00000000	0.00000000	-0.82900400

Cartesian coordinates of catatlyst

I	-1.32489900	0.00000800	0.00000200
F	-1.16928000	-1.96178000	0.12378300
C	0.80279400	-0.00001600	-0.00000200
C	1.46424400	1.21404900	0.09839700
C	1.46424900	-1.21407800	-0.09838300
C	2.85829600	1.20120300	0.10001600
H	0.90864800	2.13961600	0.16892400
C	2.85830200	-1.20122500	-0.10001400
H	0.90866600	-2.13965300	-0.16889600
C	3.55493100	-0.00000900	-0.00000400
H	3.39571400	2.14054400	0.18068800
H	3.39572300	-2.14056500	-0.18068400
H	4.64012000	-0.00000500	-0.00001100
F	-1.16917500	1.96179100	-0.12380500

Cartesian coordinates of $[PhIF]^-$

C	-3.79051300	-0.00159300	0.00017100
C	-3.08443200	1.19945200	0.00005900
C	-1.68966800	1.19108300	-0.00011500
C	-0.95220000	0.00156200	-0.00014000
C	-1.68717700	-1.18974200	-0.00013800
C	-3.08186700	-1.20119000	0.00004200
H	-3.62437400	2.14445800	0.00013700
H	-1.15685900	2.14109200	-0.00019600
H	-1.15214400	-2.13847200	-0.00013700
H	-3.61984600	-2.14732000	0.00020700
I	1.30072000	0.00025900	-0.00003700

F	3.46752200	-0.00090700	0.00025300
H	-4.87752800	-0.00273900	0.00042200

Cartesian coordinates of E-INT0

C	-4.54959000	2.71552200	-0.41228800
H	-3.89185600	3.57413900	-0.51748200
H	-5.61594900	2.90053500	-0.47244200
C	-4.06408500	1.48599400	-0.21607200
H	-4.74146100	0.64764400	-0.11686800
C	-2.62474000	1.25328300	-0.15184900
H	-2.02338800	2.14916100	-0.30353800
C	-1.93335800	0.11292300	0.02909400
C	-0.44999200	0.10394400	0.03146900
C	0.26968500	-0.93331300	-0.57607800
C	0.26227000	1.14706800	0.63353600
C	1.65811500	-0.91738400	-0.60257300
H	-0.25878200	-1.75581500	-1.04549500
C	1.65122600	1.17530800	0.61171300
H	-0.27681700	1.93545000	1.14944100
C	2.33856400	0.14070100	-0.01091800
H	2.21104300	-1.71729700	-1.08112800
H	2.19766900	1.98283600	1.08491400
C	-2.58752700	-1.23886500	0.17201500
Br	4.22902900	0.16625000	-0.04227700
F	-1.90566100	-2.02293000	1.01716000
F	-3.84571900	-1.17938600	0.62888800
F	-2.63082700	-1.88729500	-1.00893800

Cartesian coordinates of E-1,2INT1

C	1.86443900	2.09144200	1.57884900
C	1.13141600	1.01721400	2.07309600
C	-0.18435000	0.80760300	1.66195300
C	-0.72239600	1.69473200	0.74662300
C	-0.03095100	2.78435500	0.24112700
C	1.28016700	2.97492000	0.67409300
H	1.58099500	0.32083100	2.77332800
H	-0.76086300	-0.03313500	2.02146200
H	-0.50303300	3.45306800	-0.46659200
H	1.84706100	3.81691500	0.29021300
I	-2.67614300	1.30749300	-0.00731100
F	-2.50148700	3.03552300	-0.91506800
C	-3.69607200	-1.35671200	-2.49627400
C	-2.98047500	-1.80009800	-1.45689100
H	-4.77227500	-1.48218000	-2.53749800

H	-3.22459500	-0.85650300	-3.33866000
F	-2.58460900	-0.36364300	1.02846100
H	-3.47537500	-2.28179300	-0.62579300
C	-1.53503200	-1.60395300	-1.41386300
H	-1.14000300	-1.08012900	-2.28338600
C	-0.63685600	-1.92178900	-0.46196000
C	-0.96096200	-2.79013200	0.73082400
C	0.76945200	-1.44939200	-0.53422300
C	1.06494000	-0.24952400	-1.19816900
C	1.82087900	-2.11725900	0.10875200
C	2.34978600	0.27076900	-1.22333400
H	0.26690800	0.32823300	-1.65277600
C	3.11222300	-1.60150800	0.09951000
H	1.63935700	-3.04777400	0.63164300
C	3.36648000	-0.40679400	-0.56026800
H	2.55272100	1.21439200	-1.71608400
H	3.91473400	-2.12267500	0.60835000
F	-0.37686800	-4.00063900	0.58455800
F	-0.47112700	-2.27559700	1.87226900
F	-2.25678300	-3.02333800	0.92767700
Br	5.10450200	0.33782800	-0.51733000
H	2.89411000	2.23565600	1.88969000

Cartesian coordinates of E-3,4INT1

C	4.37797700	-3.99744100	0.59132200
C	3.31504800	-3.54567600	1.36749200
C	2.70489000	-2.32351900	1.09166900
C	3.19161100	-1.58258300	0.02676000
C	4.24969700	-1.99868300	-0.76511100
C	4.84156200	-3.22525300	-0.46939100
H	2.95177700	-4.14259500	2.19742300
H	1.87870700	-1.95481100	1.68394100
H	4.59880100	-1.38107300	-1.58183000
H	5.67073300	-3.57241200	-1.07686000
I	2.28393900	0.29086700	-0.42061900
F	3.67637100	0.47890000	-1.77949100
F	0.99620500	-0.17819900	1.01735500
C	-0.32556700	2.48691700	-0.87306700
C	-0.86019200	1.89869500	0.21207100
C	-0.39699300	2.29554400	1.59689900
C	-1.99755000	0.95477100	0.08777400
C	-3.09873100	1.31892500	-0.69518200
C	-1.98959900	-0.30548900	0.70003100
C	-4.17213000	0.45292900	-0.87200400

H	-3.12300600	2.30294600	-1.15326300
C	-3.05743800	-1.17736800	0.52882700
H	-1.12689000	-0.60654000	1.28117800
C	-4.14039100	-0.79061900	-0.25394200
H	-5.02686600	0.74230100	-1.47197100
H	-3.04953400	-2.15617500	0.99395500
F	-0.78938000	1.44029200	2.53977900
F	0.94134300	2.40578500	1.68513500
F	-0.89282700	3.50370800	1.92296700
Br	-5.59739600	-1.97743400	-0.47762300
H	4.84580900	-4.95061400	0.81384700
C	0.73631800	3.49078800	-0.91568500
H	1.03864000	3.96536900	0.01213700
C	1.34183000	3.81870800	-2.06198500
H	1.05848300	3.35480000	-3.00310800
H	2.13537000	4.55625400	-2.09386700
H	-0.69937700	2.15853300	-1.84252900

Cartesian coordinates of E-1,2INT2

C	2.44050626	-1.67769251	-1.92377117
C	1.76889822	-0.49547425	-2.22132904
C	0.42786913	-0.33521635	-1.87906186
C	-0.19859030	-1.38829762	-1.23348718
C	0.42706957	-2.58538535	-0.93235151
C	1.76796230	-2.71845596	-1.29208466
H	2.28817292	0.32451429	-2.70584991
H	-0.09107906	0.59160870	-2.08286321
H	-0.09278980	-3.38869924	-0.43101436
H	2.28413913	-3.64396347	-1.06075965
I	-2.19352934	-1.03374696	-0.54402711
F	-2.01009903	-2.90380789	0.39477229
C	-3.96173394	0.98912224	2.12330008
C	-3.16070282	1.69672247	1.31611714
H	-5.04113207	1.05216542	2.04573207
H	-3.56288047	0.30188817	2.86442798
F	-2.12875680	0.65077202	-1.45910319
H	-3.59223551	2.35374481	0.57357265
C	-1.71003026	1.57164496	1.41351395
H	-1.38751766	0.90861473	2.21532411
C	-0.72611098	2.06044245	0.63271235
C	-0.95604876	3.09291032	-0.44397227
C	0.67351102	1.57866874	0.75829735
C	0.91462757	0.28873010	1.25253109
C	1.76886162	2.31802127	0.28937378

C	2.18560125	-0.26557383	1.25378519
H	0.10550669	-0.34811550	1.58861843
C	3.04840024	1.77401568	0.28297479
H	1.62997731	3.32310488	-0.09010621
C	3.24396854	0.47919408	0.74878135
H	2.32542401	-1.28279311	1.60086938
H	3.88680145	2.34744823	-0.09526457
F	-0.44048521	4.28079921	-0.06556664
F	-0.33830686	2.76775282	-1.59482360
F	-2.23740521	3.31151763	-0.74311522
Br	4.96375882	-0.30243945	0.63823975
H	3.49104437	-1.78242559	-2.17277151
F	-4.13412941	-2.12673677	1.49821406
H	-3.44982523	-2.73366790	1.30668795
F	-0.16720377	-2.44868542	2.03314439
H	-0.88498506	-2.73421825	1.49476698

Cartesian coordinates of E-3,4INT2

C	4.33210700	-3.92884100	-0.01681300
C	3.59137100	-3.36816000	1.01849800
C	3.01813200	-2.10416900	0.88211500
C	3.21344400	-1.44937500	-0.32229900
C	3.95735100	-1.96506400	-1.37206900
C	4.52053700	-3.22715800	-1.20491500
H	3.45443600	-3.90550600	1.95032100
H	2.47482700	-1.65486300	1.70378400
H	4.10639500	-1.39461200	-2.28043900
H	5.10484500	-3.65864200	-2.01047300
I	2.35332600	0.46708300	-0.62936100
F	3.80753500	0.79834300	-1.84119900
F	0.87762400	-0.21919300	0.66314600
C	-0.46723800	2.51071600	-0.90150700
C	-0.98590100	1.88067300	0.16906800
C	-0.44567400	2.16593800	1.55282900
C	-2.16728300	0.99320400	0.03616400
C	-3.24776700	1.42702400	-0.74254800
C	-2.22696600	-0.27991200	0.61911000
C	-4.35815300	0.61720600	-0.94809100
H	-3.22694000	2.42330700	-1.17331100
C	-3.33152100	-1.09762600	0.41617400
H	-1.41165300	-0.65657000	1.22023400
C	-4.38830300	-0.64410500	-0.36549800
H	-5.19474100	0.96303900	-1.54364400
H	-3.36173500	-2.08635400	0.85871000

F	-0.74290300	1.20758800	2.43555400
F	0.89810300	2.29090100	1.56574100
F	-0.93914600	3.31731800	2.02864900
Br	-5.89297600	-1.75699800	-0.63620800
H	4.77069000	-4.91353400	0.10421900
C	0.62410600	3.48444600	-0.93645100
H	0.95843400	3.92611300	-0.00326700
C	1.21614400	3.83179100	-2.08517400
H	0.89953600	3.40735500	-3.03447500
H	2.02633600	4.55134700	-2.11089500
H	-0.88963200	2.25009000	-1.87127600
F	1.97107800	0.03822300	2.93390400
H	1.29549900	0.04068900	2.29077500
F	0.02169400	-2.31826800	1.72453400
H	0.34940600	-1.66607400	1.13865400

Cartesian coordinates of E-1,2TS1

C	0.32895800	-0.37208300	1.00291400
H	-0.24503700	0.49129700	0.67320000
C	-0.13350200	-1.65749100	0.61704400
H	0.50669900	-2.50662800	0.82703400
I	3.18055300	-0.30705200	-0.04396200
F	4.74176100	-0.10974100	-1.43481700
C	3.26490100	1.83138000	-0.13891100
C	2.12968100	2.58657600	0.10939400
C	4.49220900	2.40037900	-0.44404300
C	2.23386700	3.97558300	0.06009000
H	1.17252100	2.13054100	0.33029200
C	4.57441600	3.79055300	-0.48331800
H	5.34348900	1.77304200	-0.67056800
C	3.45401700	4.57643700	-0.23053600
H	1.35270300	4.57946400	0.24702100
H	5.52701800	4.25464100	-0.71488100
H	3.53014900	5.65774000	-0.26443000
C	1.61654500	-0.17993400	1.60078900
H	1.74857100	0.79015500	2.07353800
H	1.85270000	-1.01776900	2.26166500
C	-1.32726000	-1.85119300	-0.00999800
C	-1.56493800	-3.21580600	-0.63907000
C	-2.41808900	-0.88588200	-0.11102500
C	-2.68898000	-0.03438400	0.97716100
C	-3.23855600	-0.83977900	-1.25466700
C	-3.73941200	0.87352700	0.89620600
H	-2.10261500	-0.11952900	1.89775500

C	-4.27279200	0.07528400	-1.33533600
H	-3.05211800	-1.50571800	-2.08936700
C	-4.51281600	0.92900900	-0.25709000
H	-3.96450600	1.52331000	1.73376200
H	-4.89635700	0.13058800	-2.21948300
F	-0.69934700	-4.12958700	-0.20822300
F	-2.79764700	-3.65818700	-0.39653300
F	-1.42295800	-3.12083100	-1.97406500
Br	-5.93243700	2.16463100	-0.36686200
F	-0.70230600	-0.27405800	3.13475200
F	4.62576100	-2.55101600	-1.46466800
H	4.89857900	-1.66599000	-1.65281600
F	0.50860900	-2.22763000	3.15913900
H	-0.08014900	-1.37707000	3.23952800

Cartesian coordinates of E-1,4TS1

C	-0.78788500	-0.30043400	-0.69155600
H	-1.21352700	0.58749100	-0.24230400
C	0.54603700	-0.52625900	-0.59312400
H	0.99106400	-1.38496600	-1.08676300
I	-3.65038800	-1.57222900	-0.54956100
F	-5.57583500	-1.70041500	0.40932200
C	-3.88984900	0.49212600	-0.02840500
C	-3.43083100	1.46447200	-0.90225300
C	-4.45342700	0.76866400	1.20755900
C	-3.53794200	2.79675900	-0.50475300
H	-2.95170000	1.23873700	-1.84689700
C	-4.56364300	2.11042200	1.56815200
H	-4.77265000	-0.02443000	1.87329600
C	-4.11050900	3.11865200	0.72104400
H	-3.15823400	3.56794800	-1.16573100
H	-4.99771800	2.35864300	2.53073100
H	-4.19256100	4.15722200	1.02348700
C	-1.70155100	-1.12975300	-1.48682100
H	-1.91479700	-0.55953100	-2.39593900
H	-1.27096800	-2.09310300	-1.77057500
C	1.45079100	0.47212900	-0.13090700
F	1.45758100	1.85129400	-1.62447500
F	-0.79840200	1.66148900	-2.29587700
H	0.15515300	1.79056500	-2.06143100
F	-4.87682300	-1.93369100	2.65636200
H	-5.29020800	-1.93184900	1.77799700
C	0.98531300	1.59038200	0.80299400
C	2.88581300	0.14891000	-0.07599400

C	3.27078600	-1.11568100	0.38389900
C	3.86018800	1.08602200	-0.44401600
C	4.61675900	-1.43951900	0.49983300
H	2.51949000	-1.83926300	0.68447600
C	5.20343900	0.75674800	-0.35352700
H	3.53519400	2.03761900	-0.84417200
C	5.57076300	-0.49964200	0.12516300
H	4.92392800	-2.40899000	0.87294200
H	5.96606900	1.46449900	-0.65562600
F	0.71217500	1.01010000	1.98393700
F	1.93350000	2.49636300	1.00338100
F	-0.11088600	2.22529800	0.39814700
Br	7.39956300	-0.93972600	0.25921000

Cartesian coordinates of E-2,1TS1

C	1.86830500	1.37083800	1.41745700
H	2.32179700	0.99008700	2.32764700
C	0.46843600	1.10042900	1.11616500
H	0.02105700	1.94612300	0.60559300
C	0.22612500	-1.22527000	2.00038400
F	-0.04643600	-2.32484500	1.28369900
F	1.55981300	-1.19068200	2.15912500
F	-0.30974200	-1.40458200	3.21645200
C	-1.72171800	0.00413600	0.90804700
C	-2.59809500	-1.01138200	1.31853500
C	-2.22047800	1.01245100	0.06088500
C	-3.92171500	-1.03344500	0.89690400
H	-2.26044000	-1.79768900	1.98194400
C	-3.54157800	0.99506300	-0.36121300
H	-1.58394900	1.82062900	-0.29250800
C	-4.38462400	-0.03079400	0.05525900
H	-4.58925100	-1.82240400	1.22223200
H	-3.91672200	1.77511100	-1.01340300
I	3.45820400	0.23299200	-0.27102800
F	4.31278400	-1.09392500	-1.53877100
C	1.67315700	-0.27313900	-1.32258900
C	1.36219600	-1.62302900	-1.32817000
C	0.88042800	0.72602000	-1.86308100
C	0.16526900	-1.99008400	-1.94285700
H	2.01002500	-2.36168700	-0.86742700
C	-0.30591800	0.31842800	-2.47180600
H	1.08328400	1.78910700	-1.77574900
C	-0.66218200	-1.02725700	-2.51145300
H	-0.11729500	-3.03700400	-1.95568200

H	-0.95720100	1.07695800	-2.89283100
H	-1.59989800	-1.32260400	-2.96993000
C	2.45385900	2.45318000	0.75768100
H	2.01857400	2.84601700	-0.15115700
H	3.38914800	2.87781700	1.11206900
C	-0.30593500	0.02238300	1.33948300
F	1.10170700	4.06618100	1.25819400
Br	-6.17974100	-0.06080000	-0.53270000
F	3.84079500	-3.09182700	-0.15853300
H	4.17682000	-2.49307800	-0.80485900
F	0.18750800	3.36431500	-0.69215200
H	0.57909200	3.83916100	0.31310500

Cartesian coordinates of E-3,4TS1

C	-0.22947500	1.00628000	-0.33151900
I	1.65653500	-1.34977100	-0.81155800
C	2.81668400	-0.47257900	0.74830800
C	2.86027000	-1.20332700	1.92652900
C	3.45895400	0.73741000	0.56996800
C	3.57399100	-0.66576000	2.99257500
H	2.38249600	-2.17223100	2.00133500
C	4.16406300	1.25517800	1.65617600
H	3.41353500	1.27785100	-0.36574800
C	4.21646400	0.56328600	2.86055600
H	3.62431700	-1.21416900	3.92692700
H	4.67550400	2.20497000	1.54538400
C	0.57135200	0.61194700	-1.44668400
F	-0.93795900	2.99720200	-1.61084200
H	-0.07154700	0.07120800	-2.15226500
F	2.79536200	-2.96856000	-0.25461900
H	4.76562200	0.97821200	3.69877600
C	-1.53163700	0.45112600	-0.10713700
C	-2.38182400	0.29932400	-1.22141900
C	-2.01738900	0.16554600	1.18317000
C	-3.65435600	-0.21521100	-1.06361800
H	-2.06548300	0.71510600	-2.17213200
C	-3.28142600	-0.36734400	1.34092100
H	-1.38753600	0.32279200	2.05093600
C	-4.08466400	-0.56162400	0.21433200
H	-4.32647600	-0.30662500	-1.90767300
H	-3.65813100	-0.62447700	2.32319700
C	0.29890000	1.96811700	0.72961200
F	0.72207700	1.22943100	1.78657700
F	1.33468400	2.67886600	0.30278100

F	-0.64231300	2.77820500	1.16356200
Br	-5.80787500	-1.27864300	0.43958100
C	1.43993400	1.59325100	-2.17658500
H	0.91321100	2.54072900	-2.29385500
C	2.63067100	1.34575300	-2.71467500
H	3.17254000	0.41051800	-2.58590500
H	3.12044700	2.10794800	-3.31056500
F	4.69639900	-1.68576700	-1.26351600
H	4.19918400	-2.35949100	-0.83467000
F	-2.89231200	2.78258800	-0.38277000
H	-2.06491800	2.96210300	-0.93013500

Cartesian coordinates of E-1,2INT3

C	-0.90735400	0.80171800	2.08647300
H	-0.08227400	0.11608500	1.89409900
C	-0.96678900	1.83930100	1.00505200
H	-1.87318600	2.43813700	0.95247200
I	-3.02576100	-0.76727500	0.36765100
F	-3.51089900	-1.58709400	-1.61598800
C	-1.12669500	-1.55916200	-0.19459400
C	-0.27123200	-2.05965200	0.77526300
C	-0.80383200	-1.54772200	-1.54405200
C	0.98291800	-2.52504700	0.38040400
H	-0.56042600	-2.10067700	1.82025500
C	0.44479500	-2.03741700	-1.91927000
H	-1.53559300	-1.21073100	-2.26682100
C	1.34006400	-2.51193500	-0.96366200
H	1.67083100	-2.90646800	1.12750800
H	0.71631500	-2.04012900	-2.96998300
H	2.31893900	-2.87224200	-1.26378100
C	-2.20471400	0.02838300	2.23181600
H	-2.09816200	-0.76835000	2.96945200
H	-2.99330000	0.70368200	2.57941400
C	-0.00608100	2.03940800	0.09687500
C	-0.27147100	3.02495100	-1.01755200
C	1.31097500	1.35924700	0.06967600
C	2.02303200	1.15978700	1.26063600
C	1.87521900	0.92529900	-1.13458900
C	3.23701900	0.48410000	1.25976600
H	1.62116100	1.54305200	2.19430900
C	3.09554400	0.26135000	-1.14686400
H	1.34902300	1.08406200	-2.06891900
C	3.75586000	0.02774000	0.05289400
H	3.78033500	0.32077200	2.18306100

H	3.52268800	-0.08972000	-2.07927700
F	-1.31762300	3.81593900	-0.76248100
F	0.78826900	3.80698500	-1.24356600
F	-0.53859100	2.37770800	-2.16865500
Br	5.38046300	-0.93964000	0.04333400
F	-0.64398000	1.42553400	3.30351400
F	-5.65954900	-0.70593600	-1.10760900
H	-4.88941000	-1.12481300	-1.51053600

Cartesian coordinates of E-1,4INT3

C	-1.22157200	-1.67293100	-1.15358600
H	-0.69772500	-0.82948200	-1.60408000
C	-0.50723700	-2.66817300	-0.63182900
H	-0.98744600	-3.53131000	-0.17697200
I	-3.02130500	-0.03497600	0.54623300
F	-2.81785100	1.64722400	1.98143300
C	-2.05049900	1.40822900	-0.68485400
C	-2.48974300	1.56568100	-1.99120400
C	-0.97220500	2.09510500	-0.15533000
C	-1.79097800	2.44781500	-2.81307700
H	-3.35756300	1.03667800	-2.37044800
C	-0.27767500	2.95854000	-1.00155500
H	-0.68172300	1.95296200	0.87752200
C	-0.68269200	3.13315500	-2.32165400
H	-2.11996800	2.59586300	-3.83603100
H	0.59151800	3.48341500	-0.61650400
H	-0.13510600	3.80805600	-2.97069300
C	-2.69309600	-1.53782200	-1.03388800
H	-3.18382000	-1.19578200	-1.94459600
H	-3.17771900	-2.44640800	-0.66946800
C	1.00267400	-2.64771900	-0.66716800
F	1.44582200	-3.08365900	-1.90695300
F	-0.69814900	0.99171700	2.69572400
H	-1.62210600	1.32692200	2.51525400
C	1.55038200	-3.68928000	0.31181200
C	1.60372200	-1.28600400	-0.36897400
C	2.63242800	-0.80260700	-1.17525600
C	1.15870300	-0.52602100	0.71597400
C	3.19793600	0.44322400	-0.92064400
H	2.98108000	-1.39598000	-2.01196000
C	1.73452300	0.70869900	0.99444800
H	0.34716800	-0.86013800	1.35502500
C	2.73957200	1.18488300	0.16130500
H	3.98427200	0.83433700	-1.55545500

H	1.36688400	1.28369100	1.83679600
F	1.09089700	-4.90902500	0.00720200
F	2.87989900	-3.72965300	0.28168300
F	1.15783500	-3.40562300	1.55928200
Br	3.47685400	2.90009800	0.48768100

Cartesian coordinates of E-2,1INT3

C	1.46000600	-0.51460700	1.58543000
H	1.60760700	-1.53224500	1.95290400
C	0.03611800	-0.29259900	1.21013000
H	-0.38197200	0.63957100	1.58959100
C	-0.37938900	-2.32656500	-0.18971100
F	-0.12544300	-2.11456400	-1.49512300
F	0.73906000	-2.85287000	0.33774400
F	-1.32722700	-3.26371200	-0.12034200
C	-2.21481400	-0.64059000	0.26481500
C	-2.84905800	-0.85923900	-0.96489000
C	-2.93420200	-0.01217000	1.28783500
C	-4.15647900	-0.43934600	-1.17520300
H	-2.31724900	-1.35090900	-1.77186200
C	-4.24088200	0.41497100	1.08700600
H	-2.47549000	0.11928500	2.26300600
C	-4.84176600	0.20006500	-0.14802900
H	-4.64127900	-0.60424400	-2.13021500
H	-4.79497800	0.89514600	1.88487800
I	2.94568100	-0.49481500	-0.06855300
F	4.48438300	-0.05276800	-1.54585700
C	2.68517000	1.59524200	-0.36063000
C	1.41050300	2.13001900	-0.47627400
C	3.83358300	2.36950900	-0.44574700
C	1.28550700	3.50915800	-0.64241200
H	0.52945000	1.49855200	-0.46621700
C	3.68684500	3.74413600	-0.60962200
H	4.80549100	1.89321900	-0.43606800
C	2.41891300	4.31393400	-0.69834500
H	0.29715400	3.94496700	-0.74136500
H	4.57195700	4.36762700	-0.67623400
H	2.31473200	5.38581500	-0.82821300
C	1.89908500	0.47255000	2.65065500
H	1.89177300	1.49888200	2.26475700
H	2.90238100	0.23323400	3.01776500
C	-0.80645200	-1.05000300	0.48954900
F	1.01334300	0.40874100	3.70887100
Br	-6.62160100	0.77011900	-0.42872400

F	5.05626200	-2.34461400	-1.21562700
H	5.00585100	-1.42656100	-1.49843300

Cartesian coordinates of E-3,4INT3

C	-0.35866900	-1.79087200	0.46115600
I	1.05936700	1.02205100	-0.05274600
C	3.11896900	0.54773000	-0.28947000
C	4.03392500	1.47039500	0.19250200
C	3.47990500	-0.63442100	-0.91605700
C	5.38596500	1.16318100	0.05190200
H	3.70536900	2.40130800	0.64463200
C	4.83905400	-0.91536800	-1.04557200
H	2.73851300	-1.32319000	-1.30270800
C	5.78801200	-0.02158000	-0.55896700
H	6.12558800	1.86471900	0.42275100
H	5.14760100	-1.83273300	-1.53549800
C	0.65404200	-0.84363900	1.12168300
F	-0.56068300	-2.82704400	1.36031600
H	0.09975800	-0.40234700	1.95573700
F	1.79344700	2.81667900	-1.04757400
H	6.84432900	-0.24601500	-0.66287500
C	-1.69322200	-1.09803500	0.23183100
C	-2.59249300	-1.08174600	1.29999200
C	-2.02956900	-0.44325300	-0.95493200
C	-3.80843900	-0.41893200	1.19393900
H	-2.34176600	-1.60504700	2.21605300
C	-3.24643500	0.22356100	-1.06920900
H	-1.36022900	-0.45139800	-1.80709300
C	-4.12534400	0.23251300	0.00647000
H	-4.50735800	-0.40927700	2.02189100
H	-3.50972100	0.72966500	-1.99039100
C	0.13192300	-2.52311600	-0.79488500
F	0.51733700	-1.66312500	-1.75788700
F	1.18440700	-3.30109100	-0.52534800
F	-0.82775500	-3.29258100	-1.29801400
Br	-5.77558200	1.13660800	-0.14617100
C	1.87568500	-1.49103100	1.68108400
H	2.40401800	-2.20832700	1.06280600
C	2.34767800	-1.19101400	2.88904300
H	1.85070500	-0.46947100	3.53256100
H	3.25190700	-1.65213400	3.26939500
F	2.40423300	3.92725900	0.93858100
H	2.14540000	3.60712300	0.05050900

Cartesian coordinates of E-1,4TS2

C	-0.60070100	-0.27342400	0.22100600
H	-0.59534400	0.51002500	0.97237000
C	0.12097700	-0.17342400	-0.89433000
H	0.15929800	-0.98666500	-1.61745300
C	2.54160100	0.44330200	-0.74333300
C	2.72923600	-0.38674900	0.36515800
C	3.63846400	0.90241800	-1.47223900
C	4.02274400	-0.75142300	0.73450500
H	1.87894400	-0.77587900	0.93124900
C	4.92972400	0.53525900	-1.11066100
H	3.47459700	1.54518600	-2.32967000
C	5.10291200	-0.28914200	-0.00572900
H	4.18440200	-1.39777000	1.58926200
H	5.78849900	0.88122100	-1.67415800
I	-3.27362700	-1.53800400	-0.67329500
F	-5.21047000	-1.41487600	-1.79941200
C	-4.14472000	0.12554500	0.37781800
C	-5.32815400	0.63513800	-0.13669600
C	-3.52366000	0.64238600	1.50732800
C	-5.90599500	1.72450300	0.51089900
H	-5.74177900	0.13464500	-1.01026600
C	-4.13025100	1.73263400	2.13569300
H	-2.60897400	0.25170700	1.94567100
C	-5.31095100	2.27673700	1.64264400
H	-6.83159900	2.14142100	0.12427100
H	-3.65674400	2.14662900	3.02030200
H	-5.76805300	3.12680900	2.14089100
C	-1.36518000	-1.48326800	0.59800200
H	-0.93910400	-2.42304500	0.26388100
H	-1.76475800	-1.52439500	1.59595700
C	1.14247200	0.88603500	-1.15362700
Br	6.86581000	-0.80042500	0.49511200
F	0.22155500	-1.64265200	1.82653800
F	1.16389000	1.18486400	-2.51504300
C	0.83054800	2.23648000	-0.49760800
F	1.68219400	3.17097300	-0.95112500
F	0.96565000	2.18405100	0.83145500
F	-0.40439900	2.65411100	-0.77592200
F	-0.92469200	-0.13207200	3.08855700
H	-0.31840700	-0.83839400	2.58499800

Cartesian coordinates of E-INT4

C	3.24887400	-1.40168700	0.27987200

H	3.46195500	-1.21376500	1.32864800
C	2.34172800	-0.67399800	-0.35904200
H	2.12124600	-0.84002600	-1.41090400
C	0.04828200	0.19154600	0.20037100
C	-0.72570100	0.11982100	1.35613600
C	-0.56128800	0.05373500	-1.04819100
C	-2.09724400	-0.09636900	1.27185100
H	-0.25391500	0.23139800	2.32457300
C	-1.93146400	-0.15807800	-1.14210400
H	0.02449000	0.12569000	-1.95840900
C	-2.68740300	-0.23460700	0.02226900
H	-2.70432300	-0.15560000	2.16759800
H	-2.41102300	-0.26260400	-2.10821800
C	3.98860300	-2.53240600	-0.36144400
H	3.85388000	-2.52823400	-1.44860900
H	3.63459200	-3.49294700	0.03250400
C	1.54662400	0.41168000	0.31731000
Br	-4.55076000	-0.53038800	-0.09923100
F	5.33489500	-2.42983800	-0.07517100
F	1.89519000	0.47374900	1.65020700
C	1.94382300	1.78524800	-0.24184100
F	1.22655900	2.75885100	0.31692500
F	1.74003500	1.82129600	-1.56826800
F	3.23228900	2.03264800	-0.01538200

Cartesian coordinates of Z-INT0

C	-1.38272800	3.11359600	0.79427400
C	-2.40183400	2.72201000	0.02745600
H	-1.08900700	4.15753800	0.83847300
H	-0.82458900	2.41422100	1.41012000
H	-2.96058300	3.46670100	-0.53598900
C	-2.91322800	1.34755500	-0.07274400
H	-3.99148800	1.25896100	-0.18205900
C	-2.20317900	0.21079600	-0.04493700
C	-2.93346700	-1.10281400	-0.09983200
C	-0.71847400	0.10040900	-0.02424500
C	0.03348000	0.70197400	-1.03949600
C	-0.05518400	-0.60816800	0.98212200
C	1.41949000	0.61463800	-1.04665100
H	-0.47427000	1.24620500	-1.82959100
C	1.33410400	-0.70222200	0.98717100
H	-0.62106000	-1.08356100	1.77581300
C	2.05535200	-0.08673800	-0.02721600
H	1.99763300	1.08107600	-1.83627300

H	1.84538300	-1.24687800	1.77294700
F	-2.46166700	-1.88436100	-1.08515300
F	-2.78570200	-1.80334200	1.04342800
F	-4.25128500	-0.96891600	-0.29916500
Br	3.94856800	-0.20716900	-0.02745300

Cartesian coordinates of Z-1,2INT1

C	6.93126400	1.29219100	0.47310100
C	6.15143300	1.45949900	-0.66767500
C	4.97346700	0.73242300	-0.83456800
C	4.60948300	-0.15523300	0.16590400
C	5.36223900	-0.34678800	1.31389600
C	6.53580900	0.39186400	1.45834800
H	6.45382100	2.15933200	-1.43994200
H	4.35927100	0.85432600	-1.71616700
H	5.04411300	-1.04949500	2.07219600
H	7.13848700	0.25686100	2.35064100
I	2.80932500	-1.27572700	-0.05715000
F	3.28787400	-2.11411600	1.65663300
C	-0.81621400	-1.92519800	-1.03477000
C	-0.27691200	-0.87669700	-1.66126800
H	-0.80453100	-2.91300900	-1.48495000
H	-1.27734000	-1.82982100	-0.05525800
F	2.58538000	-0.27147900	-1.75824400
H	0.22581500	-1.01789800	-2.61543500
C	-0.20040900	0.48462400	-1.10776400
H	0.75337900	0.98246800	-1.26430100
C	-1.16076200	1.13930100	-0.44052900
C	-0.85081700	2.50461600	0.10918300
C	-2.55671500	0.67192300	-0.22038800
C	-3.33790100	0.27894600	-1.31319600
C	-3.11792900	0.63211900	1.05989100
C	-4.64403500	-0.15943500	-1.13681600
H	-2.91682100	0.31752100	-2.31285100
C	-4.42605000	0.19502900	1.25056500
H	-2.53238700	0.93457400	1.92097100
C	-5.17432800	-0.19892700	0.14908100
H	-5.24302900	-0.46055600	-1.98876800
H	-4.85281600	0.16194100	2.24645300
F	-1.77578100	3.40496200	-0.26071200
F	-0.83674800	2.50802300	1.45879300
F	0.33930200	2.97494100	-0.28522500
Br	-6.95709700	-0.79680300	0.40122900
H	7.84621400	1.86285800	0.59420700

Cartesian coordinates of Z-3,4INT1

C	5.02687500	-3.39285300	1.04357000
C	4.01981900	-2.85720700	1.84165300
C	3.18866600	-1.84977800	1.35363100
C	3.40007700	-1.40409600	0.05831600
C	4.39422300	-1.91269600	-0.76270100
C	5.21111900	-2.92088000	-0.25302000
H	3.87307600	-3.21899000	2.85416700
H	2.40168200	-1.42606200	1.96274900
H	4.52959300	-1.53272300	-1.76649700
H	5.99427800	-3.33423600	-0.88028900
I	2.15085700	0.13922000	-0.71324600
F	3.37263800	0.15715200	-2.25502400
F	1.09392800	-0.10932500	0.95061900
C	-0.48420600	2.44661400	-1.04921900
C	-0.92735900	1.87412100	0.08491900
C	-0.41109900	2.27289200	1.44931000
C	-2.04122000	0.88726500	0.03771700
C	-3.22636600	1.23169800	-0.62148800
C	-1.92288400	-0.39398900	0.59304900
C	-4.27781200	0.32658900	-0.72805100
H	-3.33321800	2.22646700	-1.04315900
C	-2.96639100	-1.30670500	0.49153800
H	-0.99688000	-0.68183500	1.07716100
C	-4.13503600	-0.93527500	-0.16562600
H	-5.19477300	0.60461700	-1.23498400
H	-2.86768500	-2.30044000	0.91379500
F	-0.66115500	1.34772400	2.38207900
F	0.90355200	2.51875100	1.47608800
F	-1.02347800	3.40230500	1.87474200
Br	-5.56366700	-2.17740800	-0.29691900
H	5.66837700	-4.17704100	1.43215700
C	0.51142200	3.50980800	-1.25196300
H	1.14731300	3.38024700	-2.12671000
C	0.62479800	4.62322400	-0.52612300
H	1.36873700	5.37352400	-0.77428600
H	-0.02312900	4.82689300	0.32017100
H	-0.91157000	2.04838500	-1.96964200

Cartesian coordinates of Z-1,2INT2

C	6.77122200	1.11657300	0.44263900
C	6.21806600	1.20769600	-0.83110000
C	4.95989800	0.67032900	-1.09186800

C	4.30018100	0.05083800	-0.04459100
C	4.80739000	-0.05405100	1.23841400
C	6.06709900	0.49565600	1.46962600
H	6.76281000	1.69485500	-1.63254700
H	4.50638300	0.74416400	-2.07199200
H	4.24600600	-0.50909600	2.04509100
H	6.48969100	0.43799000	2.46673600
I	2.39069700	-0.77598100	-0.47179600
F	2.83946500	-2.12706600	1.02901900
C	-0.96843100	-1.27382000	-1.66330300
C	-0.53178100	-0.06409300	-2.02749700
H	-0.88448100	-2.12355100	-2.33267500
H	-1.43087700	-1.44432300	-0.69508700
F	2.25352500	0.58879500	-1.83604700
H	-0.03882300	0.05887100	-2.98932300
C	-0.56553300	1.14199100	-1.18300400
H	0.31097500	1.77988700	-1.26147900
C	-1.54225300	1.51011700	-0.34313300
C	-1.35311200	2.75464500	0.48617300
C	-2.85664500	0.83444300	-0.17721600
C	-3.67761000	0.63509500	-1.29211800
C	-3.29983000	0.40121300	1.07589100
C	-4.90850100	0.00532200	-1.16773000
H	-3.34285700	0.97745000	-2.26609100
C	-4.53077700	-0.23413200	1.21211500
H	-2.67935100	0.55507900	1.95136800
C	-5.32347800	-0.42773900	0.08804700
H	-5.54503400	-0.14537000	-2.03168600
H	-4.87147300	-0.57530600	2.18262500
F	-2.37424900	3.60426200	0.31982900
F	-1.29958800	2.46045200	1.79929400
F	-0.22680900	3.41246700	0.18827900
Br	-6.99816200	-1.28823000	0.26630100
H	7.75217700	1.53671200	0.63692900
F	2.63770400	-1.12360700	3.31675400
H	2.62021300	-1.66193700	2.54917500
F	4.91402400	-2.91447700	-0.20099100
H	4.24130000	-2.83834200	0.44029800

Cartesian coordinates of Z-2,1TS1

C	0.68282700	-0.35682500	1.02259000
H	1.44254700	-0.75761000	1.69365400
C	0.17754400	0.98783500	1.31261800
H	0.90854000	1.63502500	1.78774300

C	-1.20794200	2.99445900	1.37358600
F	-2.35681400	3.23416500	2.00648700
F	-0.21749000	3.49262300	2.12055600
F	-1.22727800	3.70291800	0.22753900
C	-2.24883700	0.82763300	0.58027400
C	-3.09725300	1.43666100	-0.35539600
C	-2.57824900	-0.43615400	1.08418600
C	-4.23472800	0.77960400	-0.80600500
H	-2.86803100	2.42322000	-0.74210100
C	-3.70813000	-1.10284800	0.62692500
H	-1.93655400	-0.94297600	1.79835100
C	-4.52807300	-0.48871800	-0.31328800
H	-4.88910800	1.24442800	-1.53386600
H	-3.93807600	-2.09237800	1.00380000
I	2.05600400	-0.02258500	-1.16290600
F	3.39840300	0.50387400	-2.56684500
C	3.78670700	-0.77067500	-0.08874500
C	4.92442000	0.00658600	-0.23157300
C	3.68552100	-1.92418500	0.66142400
C	6.06724700	-0.44750800	0.42358400
H	4.93065200	0.93208600	-0.79282100
C	4.85433800	-2.33777700	1.30689400
H	2.76187400	-2.47928100	0.83253100
C	6.03538100	-1.61513000	1.18195100
H	6.98007200	0.13239200	0.34171900
H	4.81201800	-3.23506400	1.91449300
H	6.93253100	-1.95302700	1.68938400
C	0.18719300	-1.28709700	0.12380500
H	0.60713500	-2.28345400	0.12532600
H	-0.64673000	-1.06119400	-0.53591400
C	-1.03651900	1.51731800	1.07368600
F	-0.87434600	-2.60947900	1.69382600
Br	-6.07063900	-1.38516800	-0.93545000
F	4.12504700	2.69416800	-1.66925400
H	3.91156200	1.98532400	-2.24791300
F	1.34340700	-2.98554200	1.88749700
H	0.20866100	-2.86319600	1.88302200

Cartesian coordinates of Z-1,2TS1

C	0.59022800	-0.26423000	-1.67137000
H	1.20409900	-0.73532700	-2.42836500
C	-0.00760800	1.01044100	-1.98127700
H	0.52863800	1.63883400	-2.68713000
I	2.92679300	-0.88638200	0.00008500

F	4.79840300	-0.73445600	0.81023700
C	2.60020500	0.72586300	1.36199700
C	1.72741800	1.75478500	1.03858600
C	3.28224000	0.69551600	2.57043800
C	1.52359100	2.78080300	1.95874700
H	1.20726700	1.78311300	0.08855600
C	3.06582500	1.72711900	3.48117400
H	3.98587300	-0.09955900	2.77985800
C	2.18809000	2.76503300	3.18078200
H	0.84521500	3.58904400	1.70628000
H	3.59124900	1.71347700	4.43007800
H	2.02656800	3.56358700	3.89683000
C	0.59563600	-0.85116500	-0.39526100
H	0.06325800	-0.35787200	0.41289200
H	0.57748300	-1.93650900	-0.42990900
C	-1.18625800	1.46094000	-1.49177000
C	-1.51323800	2.92339500	-1.73893800
C	-2.19409500	0.67319600	-0.77021000
C	-2.38160300	-0.68424800	-1.08693000
C	-3.00355700	1.27218900	0.20870800
C	-3.33855800	-1.42778400	-0.41091400
H	-1.77830600	-1.17476100	-1.85091000
C	-3.95451200	0.52672900	0.88870100
H	-2.87980100	2.32018800	0.45712200
C	-4.11356200	-0.82035100	0.57339100
H	-3.48604300	-2.47324400	-0.65445100
H	-4.57110400	0.98211100	1.65419000
F	-0.76315000	3.46235200	-2.70144800
F	-2.79149800	3.11145800	-2.05719400
F	-1.26873400	3.61849500	-0.60825100
Br	-5.41322500	-1.83258400	1.48926800
F	-0.26144000	-2.25246000	-2.64481000
F	5.47579900	-2.26111900	-1.01313400
H	5.57083000	-1.71842700	-0.25592300
F	1.85425000	-2.61504000	-1.91933400
H	0.78350500	-2.54841800	-2.33773200

Cartesian coordinates of Z-1,4TS1

C	1.58078800	-2.56667500	0.97601800
H	1.61420300	-3.49050400	1.55456500
C	2.47164600	-1.63127600	1.37222500
H	3.20779900	-1.92543100	2.11759500
I	-0.42067600	-0.98126100	-0.97025000
F	-1.83013300	0.76494300	-1.27747400

C	-2.09641900	-1.60411400	0.17062400
C	-1.89331100	-2.13879200	1.43668100
C	-3.36622200	-1.44225500	-0.36788300
C	-2.99840000	-2.52773300	2.19141400
H	-0.89304300	-2.25502200	1.84541200
C	-4.45820400	-1.84322100	0.39907900
H	-3.49607200	-1.02034800	-1.35927500
C	-4.28040000	-2.38072900	1.67117000
H	-2.85162200	-2.94310700	3.18298500
H	-5.45680400	-1.73206800	-0.01002800
H	-5.14067500	-2.68684400	2.25705700
C	0.74147800	-2.67745400	-0.24869800
H	1.43459000	-2.77823700	-1.08803600
H	0.07715400	-3.53863300	-0.18610100
C	2.65515500	-0.30059500	0.82899800
F	2.24934400	-0.82378300	-1.42175600
C	4.12385200	0.05175700	0.55968700
F	4.31696000	1.32504400	0.26438800
F	4.75299200	-0.17947700	1.73640400
F	4.67634300	-0.72160500	-0.35129600
C	1.64998300	0.68750700	0.88453300
C	0.56544000	0.52403900	1.78810000
C	1.59547500	1.72327100	-0.09250800
C	-0.53591000	1.34773400	1.72392400
H	0.62184200	-0.24793600	2.54844800
C	0.46101600	2.50089700	-0.18837500
H	2.37563700	1.79246700	-0.84796800
C	-0.59957900	2.30823700	0.70571800
H	-1.36559300	1.22969400	2.41008400
H	0.36299400	3.23272800	-0.98063900
Br	-2.13388500	3.34455600	0.53532100
F	3.50860800	0.96527400	-2.20832700
H	2.98645400	0.15069400	-1.97832900
F	-2.97058100	-0.15195600	-3.10250500
H	-2.52518300	0.37038000	-2.39788200

Cartesian coordinates of Z-1,4INT3

C	1.24810100	1.75679800	1.47915200
H	1.84436800	1.41500200	2.32248200
C	-0.06061400	1.85569300	1.73644700
H	-0.38289500	1.60250300	2.74400300
I	2.17949100	0.23698900	-1.07080500
F	2.31197900	-1.68980800	-2.10116300
C	2.97315000	-0.95630200	0.51419700

C	3.77309900	-0.36748200	1.47852500
C	2.64260200	-2.30148900	0.52899700
C	4.24602200	-1.16379200	2.52053400
H	4.05428900	0.67806100	1.42919200
C	3.11808500	-3.07643800	1.58364800
H	2.07224300	-2.72312100	-0.28832800
C	3.91231600	-2.51260500	2.57800300
H	4.88411100	-0.72234400	3.27855900
H	2.87097900	-4.13216600	1.61559900
H	4.28231700	-3.12745100	3.39137600
C	2.03638500	1.99904500	0.24252400
H	1.60674200	2.74610800	-0.42091600
H	3.05166100	2.31198300	0.48466500
C	-1.26854100	2.22055300	0.91290700
F	-1.98594900	3.14782500	1.65568300
C	-1.01306300	2.95177200	-0.40610100
F	-2.16037700	3.36986200	-0.93380000
F	-0.22668700	4.01934500	-0.23696200
F	-0.42273600	2.13858400	-1.29921200
C	-2.15965500	1.01695500	0.65382600
C	-3.54401800	1.17932500	0.67996500
C	-1.61241700	-0.22856900	0.35267200
C	-4.37997400	0.10052500	0.41649400
H	-3.96536300	2.15029900	0.91246200
C	-2.43981200	-1.31239800	0.08094500
H	-0.53507900	-0.36012400	0.33882700
C	-3.81852600	-1.13640800	0.11769700
H	-5.45718200	0.21569300	0.44034700
H	-2.02050100	-2.28341100	-0.15420400
Br	-4.95081400	-2.60626400	-0.24114500
F	4.68120100	-1.80491500	-1.72173800
H	3.76406000	-1.89136000	-1.98919500

Cartesian coordinates of Z-1,2INT3

C	-0.98237400	1.17190400	-1.99884700
H	-2.04552900	1.21299500	-2.26259900
C	-0.75138400	2.19098900	-0.90285500
H	-1.52563500	2.94523000	-0.80242600
I	-1.14266200	-1.08575800	0.19279000
F	-1.85811300	-1.80284300	2.23274600
C	-3.24374100	-0.98494000	-0.19419800
C	-3.71501300	-0.96591200	-1.49891400
C	-4.08295300	-0.92285700	0.90835900
C	-5.09026000	-0.86494800	-1.70424300

H	-3.05113300	-1.04064600	-2.35198300
C	-5.45353400	-0.82245700	0.67789400
H	-3.66033100	-0.98763300	1.90382800
C	-5.95736600	-0.78876000	-0.61914500
H	-5.47553600	-0.85301000	-2.71818200
H	-6.12735600	-0.77310200	1.52658300
H	-7.02633500	-0.71004300	-0.78542000
C	-0.60116300	-0.28116800	-1.73704200
H	-0.98586500	-0.91684700	-2.53517900
H	0.48515400	-0.38981500	-1.73614800
C	0.30513800	2.27489300	-0.09302900
C	0.35719500	3.37174700	0.94253800
C	1.47346200	1.35818500	-0.10591300
C	2.30760800	1.30594500	-1.22659900
C	1.69146700	0.48323600	0.96360600
C	3.34055100	0.37647800	-1.28827000
H	2.12323700	1.97394100	-2.06168600
C	2.70100700	-0.47271300	0.89359100
H	1.04818700	0.47995900	1.83723100
C	3.52011400	-0.51117800	-0.22989400
H	3.99263300	0.32876700	-2.15244200
H	2.81902600	-1.18211700	1.70432600
F	-0.69247900	4.19939900	0.87399900
F	0.38282700	2.85832500	2.18056000
F	1.46446900	4.10739900	0.79201700
Br	4.89726700	-1.80255700	-0.33485900
F	-0.27256400	1.56228300	-3.13008400
F	0.34077500	-1.30371400	2.87766800
H	-0.61055300	-1.55129300	2.77188700

Cartesian coordinates of Z-2,1INT3

C	1.31932000	0.38120300	-1.83124700
H	1.94667600	0.62893400	-2.69458700
C	0.82381300	1.64542400	-1.24105700
H	1.53846200	2.46515500	-1.28031400
C	-0.54935100	3.25918200	-0.01766600
F	-0.77399700	3.14821100	1.30466200
F	0.50557600	4.07437100	-0.16531300
F	-1.61362800	3.87486200	-0.54582500
C	-1.49375500	0.97776100	-0.43737700
C	-1.61459700	0.23577300	0.74033100
C	-2.48228300	0.87418700	-1.41849400
C	-2.68452400	-0.63422900	0.92007600
H	-0.86720100	0.33477100	1.51951400

C	-3.55841500	0.01061300	-1.24659900
H	-2.39273500	1.45325200	-2.33042600
C	-3.64470400	-0.74135400	-0.07991800
H	-2.77432500	-1.22014400	1.82744300
H	-4.32175500	-0.08171000	-2.01007800
I	2.84396400	-0.66455800	-0.56711600
F	4.00156100	-1.78736900	0.88471900
C	1.84905000	-0.24870000	1.25565000
C	1.74923800	1.05351300	1.72407800
C	1.40968600	-1.34214600	1.99130600
C	1.14143500	1.26817200	2.96155500
H	2.13270200	1.89302400	1.15609400
C	0.80339400	-1.10953900	3.22201100
H	1.57575200	-2.34970900	1.63062000
C	0.66529000	0.19185400	3.70358400
H	1.04262400	2.28155700	3.33401800
H	0.45279600	-1.95075000	3.81025500
H	0.19498800	0.36563400	4.66549900
C	0.30281900	-0.65984500	-2.25978500
H	-0.29785600	-1.01509700	-1.41671300
H	0.81159100	-1.51331100	-2.72089800
C	-0.33894800	1.89994200	-0.62983100
F	-0.53774700	-0.10496800	-3.20257300
Br	-5.09987300	-1.92694000	0.15484000
F	5.26609300	-2.39507400	-1.03625900
H	4.90823200	-2.28826400	-0.14708100

Cartesian coordinates of Z-1,2TS2

C	-0.13896300	1.88263500	-0.02678700
H	0.50654000	2.75782400	-0.07418900
I	3.71732200	0.66836500	0.32911600
F	5.73566300	0.42769000	-0.53878800
C	3.54626400	-1.38492500	-0.28616500
C	2.43224000	-2.13361300	0.07244100
C	4.58835700	-1.90190900	-1.04169200
C	2.37383700	-3.45897700	-0.36145000
H	1.61387900	-1.76878800	0.68634300
C	4.49807900	-3.22577000	-1.46680200
H	5.42790100	-1.24255200	-1.24588900
C	3.39471700	-4.00545000	-1.13182300
H	1.51069200	-4.05559600	-0.08410500
H	5.30238800	-3.64679500	-2.06329800
H	3.33174700	-5.03698000	-1.46595000
C	1.66111000	0.64903100	1.21901500

H	1.69497900	1.58463600	1.76699000
H	1.70124300	-0.19466000	1.88807400
C	-1.44775800	2.06125400	-0.22369300
F	-0.02674500	0.68689600	2.39538400
C	-1.94405500	3.45092900	-0.51098300
F	-3.02561900	3.75840600	0.22228400
F	-2.31565600	3.59588800	-1.80490400
F	-1.02701100	4.40164800	-0.27872300
C	0.59366700	0.57849100	0.12415300
H	-0.06225500	-0.26570200	0.32195000
F	1.16766300	0.35788000	-1.13795600
C	-2.49261000	1.00403300	-0.18108000
C	-2.51726100	0.10090600	0.88648100
C	-3.46137700	0.89982200	-1.18687200
C	-3.46874200	-0.91316300	0.93121600
H	-1.78033600	0.20842000	1.67723200
C	-4.42663100	-0.09939000	-1.14060300
H	-3.45822400	1.59696500	-2.01726900
C	-4.41382800	-0.99918200	-0.08126000
H	-3.46358500	-1.62661200	1.74668600
H	-5.17486500	-0.18568300	-1.92007200
Br	-5.72890100	-2.37332400	-0.02373900
F	-0.08989200	-1.55967100	2.06395400
H	-0.08451200	-0.52847700	2.31538100

Cartesian coordinates of Z-2,1TS2

I	2.23172600	0.62433600	-0.92547300
F	3.66120100	1.26278200	-2.46452100
C	3.91360300	-0.58959600	-0.32452900
C	5.13689500	-0.27688800	-0.90216600
C	3.76444500	-1.59044900	0.62679000
C	6.25838100	-0.99830000	-0.49962000
H	5.15019900	0.50076500	-1.65933800
C	4.90818700	-2.29360400	1.01160500
H	2.81834800	-1.87465200	1.07464800
C	6.14996400	-2.00313700	0.45816800
H	7.22299900	-0.76889800	-0.94342000
H	4.80265000	-3.07737200	1.75509000
H	7.02950200	-2.55985600	0.76865500
F	-0.03713500	-0.64022800	2.59752300
C	0.85316600	-0.15767800	0.86446600
H	1.64092700	-0.38975600	1.56006600
C	0.21253900	1.14719800	1.08043600
H	0.87960400	1.87661500	1.53134600

C	-1.00505400	1.59710600	0.72836800
C	-1.31626300	3.04933600	0.92842000
C	-2.16982500	0.77576500	0.30768700
C	-2.93048000	1.09272500	-0.82309800
C	-2.55275500	-0.31122100	1.10651600
C	-4.04398500	0.33317400	-1.16823000
H	-2.64642900	1.93619900	-1.44238000
C	-3.66259200	-1.07695100	0.76558400
H	-1.93890000	-0.55248900	1.97366300
C	-4.39624000	-0.74506500	-0.36722500
H	-4.62867700	0.57179500	-2.04905700
H	-3.95789200	-1.92309900	1.37549700
F	-1.58602500	3.67338000	-0.24441100
F	-2.40667600	3.23242800	1.69502300
F	-0.31806000	3.74145300	1.49791800
Br	-5.91796800	-1.78712200	-0.83280000
C	0.17080900	-1.34005700	0.19814600
H	-0.35796000	-1.93515900	0.93778000
H	-0.51436700	-0.99979200	-0.58590700
F	1.11276000	-2.15983000	-0.40354200
F	1.44686200	-2.37629900	2.57380100
H	0.74626500	-1.62212100	2.68740000

Cartesian coordinates of Z-INT4

C	2.57439600	-0.30796200	0.00633300
H	3.61362600	-0.01367200	0.11027700
C	0.15823900	0.37805000	0.01529200
C	-0.38871800	-0.68912300	0.73915500
C	-0.70046100	1.21053000	-0.71435300
C	-1.75617300	-0.93814100	0.71590200
H	0.26198300	-1.31993200	1.33620900
C	-2.06771500	0.96912800	-0.74247800
H	-0.29924000	2.04998700	-1.27035800
C	-2.58389700	-0.10682100	-0.02848500
H	-2.17804800	-1.76211400	1.27951800
H	-2.73088300	1.60930000	-1.31239500
C	3.45199500	-2.43284600	-0.96360500
H	3.45742100	-2.08146000	-2.00106600
H	3.32515200	-3.51954000	-0.94124200
C	1.61980300	0.62608000	0.05549500
Br	-4.44482600	-0.43526300	-0.06074700
F	4.65685800	-2.11446300	-0.37702300
C	2.04420800	2.07220500	0.17267200
F	1.32093400	2.72082400	1.09130000

F	1.86845500	2.72187900	-0.99591800
F	3.33313300	2.20519100	0.49924100
C	2.32497000	-1.77446600	-0.19925800
F	2.22923700	-2.40307500	1.03982600
H	1.38147900	-1.94138400	-0.73164300

Cartesian coordinates of E-1,4TS1-w/o HF

C	1.03311700	0.96158500	1.04546500
H	1.38237600	1.76798800	0.40531300
C	-0.29869000	0.70743300	1.10714800
H	-0.66381200	-0.09949000	1.73769400
I	2.80599800	-1.51461000	0.37372900
F	3.76310400	-2.87553900	-0.76869300
C	4.51231500	-0.32469800	-0.14484400
C	4.43965300	1.05880500	-0.11812500
C	5.66846800	-1.00563100	-0.49449100
C	5.58017200	1.79061500	-0.44575800
H	3.52423500	1.58139400	0.12906100
C	6.79915300	-0.25523800	-0.80995500
H	5.66103600	-2.08600500	-0.54210500
C	6.75955100	1.13577900	-0.78406600
H	5.53414500	2.87424600	-0.43967100
H	7.71476700	-0.77043200	-1.08032900
H	7.64491400	1.70987800	-1.03506800
C	2.03759200	0.15977200	1.72950600
H	2.91833400	0.71669400	2.04767500
H	1.62447900	-0.40638600	2.56672600
C	-1.30228500	1.41092400	0.37258000
F	-1.91138400	2.91922000	1.73674900
C	-0.92037400	2.47293200	-0.64074200
C	-2.61939000	0.76738000	0.17846000
C	-2.95414800	0.21911800	-1.06108300
C	-3.49303500	0.65743300	1.26219000
C	-4.15540800	-0.46550600	-1.21275500
H	-2.27922000	0.31648100	-1.90534400
C	-4.69484800	-0.02040200	1.11130100
H	-3.22498000	1.17515900	2.17607300
C	-5.01012100	-0.58258800	-0.12303800
H	-4.42704000	-0.90153400	-2.16665400
H	-5.39198400	-0.10113000	1.93693000
F	-0.34321500	1.84610700	-1.69759000
F	-1.97433900	3.12862900	-1.09436800
F	-0.03335600	3.34810800	-0.17836200
Br	-6.64379300	-1.51221600	-0.32709600

Cartesian coordinates of E-1,4TS1-1HF

C	-0.91111600	0.27827800	-1.35650900
H	-1.19950400	1.25891700	-0.95647600
C	0.41430400	-0.16828900	-1.09118500
H	0.90356600	-0.79580100	-1.83224400
I	-2.87533500	-1.52758200	-0.19157400
F	-4.05721000	-2.41676400	1.07848000
C	-4.32308300	0.05029800	-0.11118400
C	-5.64416800	-0.37136400	-0.06650900
C	-3.91222200	1.36786700	-0.07974000
C	-6.62263600	0.61696500	-0.01887100
H	-5.89775400	-1.42376500	-0.03789900
C	-4.92306300	2.33251300	-0.03801900
H	-2.87824700	1.71837000	-0.05283600
C	-6.26307100	1.96429800	-0.01470100
H	-7.66708500	0.32612100	0.01553400
H	-4.62252900	3.37449200	-0.01492700
H	-7.03424300	2.72659000	0.01886000
C	-1.82827700	-0.40461900	-2.14687900
H	-2.69302100	0.13577900	-2.52339700
H	-1.51715200	-1.27666900	-2.72030800
C	1.15856600	0.26515700	-0.03007300
F	-1.53027100	2.91702500	-0.39311900
C	0.52193300	0.60284500	1.30718000
F	1.21649100	1.46573800	2.02884500
F	-0.74438900	0.99827500	1.24036000
F	0.50916800	-0.56790600	2.00833200
C	2.61751300	0.11237700	-0.02304000
C	3.31127900	0.42473700	-1.19965300
C	3.33857100	-0.29928000	1.10700600
C	4.69190400	0.30125700	-1.26462700
H	2.75818400	0.81410600	-2.04789100
C	4.71775200	-0.43682000	1.04643600
H	2.82154900	-0.53129200	2.03033800
C	5.38236600	-0.13921300	-0.13999900
H	5.23125600	0.55706900	-2.16881800
H	5.27829300	-0.76903300	1.91194600
Br	7.26007700	-0.32086200	-0.21714700
F	0.69816000	2.52502500	-0.27905900
H	-0.38009200	2.80256600	-0.30067000

Cartesian coordinates of E-1,4TS1-3HF

C	-0.75188200	0.78832600	-0.63821300

H	-1.03620300	1.63604900	-0.02201700
C	0.54971100	0.25786800	-0.55166000
H	0.87007500	-0.40590900	-1.35211500
I	-2.77078800	-1.26170200	-0.07019300
F	-3.90416500	-2.62072000	0.95624700
C	-4.66419800	-0.26360500	-0.30627800
C	-5.77486900	-1.09139000	-0.36512000
C	-4.71198400	1.11581000	-0.37333700
C	-7.01651100	-0.47945800	-0.51724100
H	-5.67973300	-2.16435300	-0.26587400
C	-5.97291700	1.69573600	-0.52866100
H	-3.83355900	1.75537400	-0.33323300
C	-7.11562600	0.90721600	-0.60206000
H	-7.90575400	-1.09843500	-0.56885400
H	-6.03896700	2.77655200	-0.58856000
H	-8.08793000	1.37275600	-0.72246100
C	-1.70767300	0.28756400	-1.54630800
H	-2.48582500	0.99812800	-1.81548700
H	-1.35709200	-0.37812400	-2.33276700
C	1.52414300	0.68061300	0.31423500
F	1.68591000	3.05839000	-0.27299000
C	1.22552400	1.33348800	1.66170300
F	2.22782400	2.06280900	2.12911100
F	0.11805600	2.05955600	1.70440100
F	1.05906600	0.29422900	2.50697500
C	2.91618300	0.26855900	0.10531100
C	3.94214600	1.22574600	0.13245200
C	3.21712400	-1.07145400	-0.17263700
C	5.25096000	0.83797400	-0.11444300
H	3.68277400	2.26611900	0.28677600
C	4.52985100	-1.46444700	-0.39179700
H	2.42644000	-1.81544300	-0.17424900
C	5.53537900	-0.50247700	-0.36314000
H	6.04918800	1.57057400	-0.11961300
H	4.77440800	-2.50278800	-0.58064800
Br	7.32035500	-1.02710700	-0.67436600
F	-0.53617600	3.65005000	-0.26269100
H	0.70730200	3.36416500	-0.30218900
F	-1.75658200	-3.84951600	1.11466300
H	-2.66985900	-3.67221400	1.24293700
F	-2.55824100	2.96873500	-1.06793100
H	-1.64934300	3.34832400	-0.75555400

Cartesian coordinates of E-1,2TS1-w/o HF

C	-0.94527700	-0.01706500	-0.64175700
H	-1.41507200	0.74424600	-0.03228600
C	0.48259800	-0.20189200	-0.53341900
H	0.86744200	-1.13898200	-0.92817900
I	-3.41967400	-1.77645000	0.12472800
F	-5.01292600	-2.25710500	1.28753200
C	-4.40226900	0.15574400	-0.05092500
C	-3.88672000	1.16070800	-0.85734700
C	-5.56220200	0.29683300	0.69685300
C	-4.59218600	2.36706300	-0.90062300
H	-2.97190300	1.11527500	-1.44838500
C	-6.24008500	1.51153600	0.62591100
H	-5.91568200	-0.52586300	1.30308700
C	-5.75968900	2.54677900	-0.17009700
H	-4.19684700	3.16399000	-1.52152000
H	-7.15112900	1.63842700	1.20140600
H	-6.29291600	3.49044200	-0.21840200
C	-1.78379100	-1.04785500	-1.23765200
H	-2.28306900	-0.61818600	-2.10703900
H	-1.22113500	-1.93056400	-1.55103600
C	1.38594700	0.66470900	-0.02606400
C	1.00262000	1.99693400	0.59616700
C	2.82675600	0.34955700	-0.02022800
C	3.65113200	0.75121600	1.04127200
C	3.39277300	-0.37349200	-1.07849600
C	4.99601600	0.41075000	1.05997900
H	3.23782000	1.31702900	1.86835600
C	4.73954000	-0.70957200	-1.07256200
H	2.78281100	-0.64052600	-1.93530200
C	5.53077500	-0.31979500	0.00299800
H	5.62892500	0.71029400	1.88673700
H	5.17702600	-1.25635000	-1.89928800
F	0.95373900	1.86643800	1.93733900
F	1.91874000	2.92852400	0.32259200
F	-0.17527600	2.45938900	0.19411400
Br	7.36185800	-0.77295000	0.02067900
F	-1.20474400	1.43419500	-2.24785900

Cartesian coordinates of E-1,2TS1-1HF

C	1.29085800	1.36243900	1.50843400
H	1.42596900	2.42722600	1.32357400
C	-0.00817300	0.80857600	1.18137000
H	-0.22346800	-0.16876300	1.60417800
I	2.50610400	-1.40618600	0.60749200

F	2.82932000	-2.96560000	-0.56455300
C	3.59957000	-0.39726700	-0.92568700
C	3.34248500	0.93067400	-1.22151100
C	4.55831600	-1.13960500	-1.60040300
C	4.08157800	1.54218900	-2.23263600
H	2.56831100	1.48967300	-0.70987900
C	5.29544000	-0.50796300	-2.59913400
H	4.69817900	-2.18702900	-1.36797000
C	5.06199200	0.82787200	-2.91365100
H	3.87957900	2.57763600	-2.48472700
H	6.05337800	-1.07106800	-3.13318500
H	5.63925500	1.31029200	-3.69503100
C	2.43246400	0.60496500	1.82274500
H	3.37647300	1.14098700	1.85732000
H	2.21447300	-0.05655600	2.67335400
C	-0.90864100	1.42612000	0.39048100
C	-0.60593000	2.80135200	-0.17758300
C	-2.19405700	0.78391000	0.05619300
C	-2.23529000	-0.59554700	-0.18223700
C	-3.39243700	1.51070500	0.02622200
C	-3.43727700	-1.23969500	-0.44192600
H	-1.31299700	-1.16793900	-0.18434200
C	-4.60016100	0.87398500	-0.22252600
H	-3.38870400	2.57691200	0.22223700
C	-4.61287000	-0.49735900	-0.45858200
H	-3.46334400	-2.30545400	-0.63482500
H	-5.52742000	1.43437500	-0.23071600
F	0.59675600	2.81762000	-0.79442200
F	-1.50003400	3.19828000	-1.08240700
F	-0.55611000	3.73289600	0.78270600
Br	-6.25242800	-1.36339000	-0.81215800
F	0.44621900	1.13120600	3.59159700
F	0.79641700	-1.06652000	3.11781500
H	0.55144700	0.04316300	3.47441000

Cartesian coordinates of E-1,2TS1-3HF

C	1.37767700	1.69001300	-0.78132000
H	1.46073300	2.74364700	-1.02147400
C	0.12841500	1.04010700	-0.66847400
H	0.13968600	-0.04442600	-0.63903900
I	2.76518200	-1.16200400	-0.90574800
F	2.85616700	-3.36931200	-0.85066600
C	2.45197600	-1.39283300	1.21894500
C	2.02334800	-0.31419500	1.97642200

C	2.71317400	-2.64625600	1.75290000
C	1.85226900	-0.51704900	3.34737600
H	1.80977000	0.67375600	1.58441400
C	2.53621400	-2.81346300	3.12469100
H	3.02782800	-3.45548700	1.10749100
C	2.10851000	-1.75591000	3.92206000
H	1.51721900	0.31836300	3.95273600
H	2.73912500	-3.78432500	3.56429400
H	1.97574800	-1.89787700	4.98933000
C	2.66562400	0.99919900	-0.76759900
H	3.26821700	1.42870700	-1.57865500
H	3.20502700	1.38219900	0.11698200
C	-1.07725800	1.64815200	-0.45542600
C	-1.23081400	3.15441100	-0.23372100
C	-2.30298300	0.86237800	-0.33606500
C	-2.40585200	-0.37619100	-0.99712000
C	-3.39408200	1.30794400	0.43422800
C	-3.54916800	-1.14861200	-0.89063600
H	-1.60047400	-0.72230200	-1.63623000
C	-4.53333300	0.53024000	0.56009400
H	-3.34468700	2.25296200	0.96031300
C	-4.60603700	-0.69241100	-0.10390700
H	-3.62961100	-2.09289800	-1.41533900
H	-5.36472600	0.86679700	1.16752300
F	-0.22420400	3.88207200	-0.69971000
F	-2.33974100	3.58035100	-0.85447300
F	-1.37193100	3.39984300	1.06829100
Br	-6.15815800	-1.73935700	0.05258200
F	1.25639400	2.60075100	1.28321400
F	3.44080800	3.11763500	1.09365500
H	2.33329100	2.92975600	1.27737600
F	3.23705700	3.73325200	-1.19028900
H	3.41153000	3.58640900	-0.23948200
F	2.77520400	-3.13615600	-3.22003800
H	2.81833700	-3.45064100	-2.31052200

Cartesian coordinates of a

C	-0.15253500	-0.27887500	-1.98094600
H	0.53464100	0.54053400	-1.77146000
C	0.31330600	-1.58962300	-1.76349400
H	-0.33018000	-2.42984100	-2.00578400
I	-2.09565400	-0.07351300	0.27646200
F	-3.03403700	-0.09232300	2.01454600
C	-3.65545500	1.23027600	-0.28921300

C	-3.35649400	2.51819800	-0.71915800
C	-4.96426600	0.77684000	-0.17564800
C	-4.39956800	3.36946800	-1.07183100
H	-2.32837400	2.86427500	-0.77008800
C	-5.99745800	1.64314300	-0.52669600
H	-5.17326600	-0.22128500	0.19632600
C	-5.71779100	2.92981900	-0.97810800
H	-4.17998500	4.37742200	-1.40695700
H	-7.02479600	1.30519600	-0.44326000
H	-6.52927600	3.59618200	-1.25064900
C	-1.48811800	0.02708500	-2.20388700
H	-1.77364300	1.04639400	-2.44211700
H	-2.16343800	-0.75322400	-2.54630100
C	1.30799200	-1.77018200	-0.84410900
F	-0.07189600	-1.69671500	0.79557800
C	1.66369200	-3.17724600	-0.41174000
C	2.30742200	-0.72618100	-0.48824400
C	3.37616600	-0.54398500	-1.37265500
C	2.21994600	0.04705800	0.67145100
C	4.35310600	0.40895700	-1.10924800
H	3.44571200	-1.15487800	-2.26708000
C	3.19490500	1.00524700	0.93286400
H	1.39448000	-0.08761800	1.35948100
C	4.24997200	1.17757700	0.04461800
H	5.18318600	0.55471000	-1.79019600
H	3.13238500	1.61323700	1.82785100
F	0.64788600	-4.02568000	-0.54972400
F	2.66000800	-3.60687300	-1.21622400
F	2.10798000	-3.22625900	0.83469000
Br	5.56873000	2.48502900	0.40450300
F	-0.18581200	-0.40515700	2.73263800
H	-0.08287900	-1.08935800	2.03531300
F	-4.71512400	-1.86302600	1.42018200
H	-4.15044200	-1.28271400	1.89590200

Cartesian coordinates of c

C	-0.11674000	0.56921000	0.87483000
I	2.75542600	-0.38883700	0.09245200
F	3.61677300	0.12964900	1.80172000
C	4.81352100	-0.82728200	-0.28299900
C	5.19050600	-2.12570300	-0.60314100
C	5.73413600	0.21907000	-0.28635300
C	6.52011900	-2.38459800	-0.93427300
H	4.46602100	-2.93480900	-0.58928400

C	7.05905600	-0.05145400	-0.60876700
H	5.41780600	1.22590500	-0.02865900
C	7.45006600	-1.35010900	-0.93505900
H	6.82604600	-3.39531300	-1.18297000
H	7.78720600	0.75288400	-0.60902600
C	1.09991200	0.64688000	1.57854400
H	1.59201300	1.61416900	1.64265800
H	1.22800800	-0.01731800	2.42635700
F	0.30408200	-0.46796200	-0.89476400
F	3.84934600	2.53353400	1.15834000
H	3.91438900	1.73617300	1.64617500
F	-0.44807600	-2.28637100	0.38096900
H	-0.10008700	-1.61693900	-0.24596600
C	-0.64140200	1.74064800	0.21819300
H	0.04078200	2.57741500	0.09942600
C	-1.93375900	1.90755200	-0.14301300
H	-0.76923700	-0.27483600	1.07033900
C	-2.35015500	3.29418400	-0.59372300
C	-2.99593600	0.88918700	-0.11094800
C	-2.71306100	-0.42488100	-0.50589000
C	-4.29801000	1.21540800	0.30150800
C	-3.69110300	-1.40881700	-0.44097400
H	-1.72588400	-0.66250200	-0.88235700
C	-5.28115700	0.23923500	0.35734200
H	-4.54085400	2.22910400	0.59869200
C	-4.96636000	-1.06820300	-0.00765400
H	-3.45586300	-2.42733200	-0.72518800
H	-6.28442700	0.48358600	0.68549300
F	-3.01544700	3.93030400	0.38862100
F	-3.16203300	3.23865500	-1.65115400
F	-1.30592800	4.06184700	-0.91906700
Br	-6.30698200	-2.39618100	0.07819900
H	8.48460300	-1.55440300	-1.19034500

Cartesian coordinates of e

C	-1.53799900	2.08775200	-2.47957000
I	-2.25343400	-0.22431200	-0.56322000
F	-0.61526000	-0.30175000	0.44984700
C	-2.59660000	-2.05187500	0.46123700
C	-3.70912900	-2.21625200	1.27696400
C	-1.68525400	-3.08334800	0.24147700
C	-3.91492000	-3.44999000	1.89198000
H	-4.40345500	-1.39874700	1.44554200
C	-1.89448500	-4.30402600	0.87288700

H	-0.82029100	-2.92172700	-0.39631500
C	-3.00931900	-4.48738900	1.69072200
H	-4.77724200	-3.59368400	2.53412400
H	-1.18946700	-5.11460000	0.72216400
C	-0.64206800	1.67414600	-1.50136900
H	0.04918300	0.87104500	-1.74626600
F	-3.40903100	1.38687000	-2.24611700
F	0.74581100	-1.39983300	-1.38398600
H	0.79070200	-0.97805000	-0.55360400
F	-3.35687800	2.99824900	-0.53935700
H	-3.52613000	2.36016300	-1.26647200
C	-0.36546400	2.51836100	-0.34497700
H	-1.12029700	3.26804300	-0.11938500
C	0.69824500	2.37713900	0.46317200
C	1.76196200	1.36301200	0.26327200
C	2.03425000	0.39679500	1.24245300
C	2.48740500	1.32716000	-0.93285600
C	2.99350400	-0.58806400	1.02276900
H	1.47914400	0.40906900	2.17333900
C	3.44647800	0.34753400	-1.16360500
H	2.29300900	2.07959900	-1.69100800
C	3.68884500	-0.60608700	-0.18187400
H	3.19877200	-1.33735200	1.77841000
H	4.00076200	0.32181200	-2.09430300
C	0.83190400	3.25222600	1.67294000
F	0.70079600	2.54355700	2.81545600
F	-0.08501300	4.22423300	1.71152400
F	2.04071900	3.83230800	1.72678500
H	-2.00149500	3.06292600	-2.40636100
H	-1.54918500	1.60478900	-3.44808600
H	-3.17119400	-5.44501600	2.17423800
Br	4.98470700	-1.94842900	-0.48675500
